# Risk stratification using coronary artery calcium scoring based on low tube voltage computed tomography

**DOI:** 10.1007/s10554-022-02615-x

**Published:** 2022-04-23

**Authors:** Fabiola A. Bechtiger, Marvin Grossmann, Adam Bakula, Dimitri Patriki, Elia von Felten, Tobias A. Fuchs, Catherine Gebhard, Aju P. Pazhenkottil, Philipp A. Kaufmann, Ronny R. Buechel

**Affiliations:** Department of Nuclear Medicine, Cardiac Imaging, University Hospital and University Zurich, Zurich, Switzerland

**Keywords:** Coronary artery calcium scoring, Computed tomography, Low-dose, Cardiovascular imaging

## Abstract

To determine if coronary artery calcium (CAC) scoring using computed tomography at 80 kilovolt-peak (kVp) and 70-kVp and tube voltage-adapted scoring-thresholds allow for accurate risk stratification as compared to the standard 120-kVp protocol. We prospectively included 170 patients who underwent standard CAC scanning at 120-kVp and 200 milliamperes and additional scans with 80-kVp and 70-kVp tube voltage with adapted tube current to normalize image noise across scans. Novel kVp-adapted thresholds were applied to calculate CAC scores from the low-kVp scans and were compared to those from standard 120-kVp scans by assessing risk reclassification rates and agreement using Kendall’s rank correlation coefficients (Τ_b_) for risk categories bounded by 0, 1, 100, and 400. Interreader reclassification rates for the 120-kVp scans were assessed. Agreement for risk classification obtained from 80-kVp and 70-kVp scans as compared to 120-kVp was good (Τ_b_ = 0.967 and 0.915, respectively; both p < 0.001) with reclassification rates of 7.1% and 17.2%, respectively, mostly towards a lower risk category. By comparison, the interreader reclassification rate was 4.1% (Τ_b_ = 0.980, p < 0.001). Reclassification rates were dependent on body mass index (BMI) with 7.1% and 13.6% reclassifications for the 80-kVp and 70-kVp scans, respectively, in patients with a BMI < 30 kg/m^2^ (n = 140), and 2.9% and 7.4%, respectively, in patients with a BMI < 25 kg/m^2^ (n = 68). Mean effective radiation dose from the 120-kVp, the 80-kVp, and 70-kVp scans was 0.54 ± 0.03, 0.42 ± 0.02, and 0.26 ± 0.02 millisieverts. CAC scoring with reduced tube voltage allows for accurate risk stratification if kVp-adapted thresholds for calculation of CAC scores are applied.

*ClinicalTrials.gov* NCT03637231.

**Supplementary Information:**

The online version contains supplementary material available at 10.1007/s10554-022-02615-x.

## Introduction

The coronary artery calcium (CAC) score provides an estimate of the degree of coronary atherosclerotic disease and has been shown to correlate strongly with the overall amount of coronary plaque burden [1]. Moreover, the CAC score has proven to be a highly valuable tool for the evaluation of an individual's future cardiovascular risk. Several studies in many thousand subjects have consistently shown that CAC scoring provides accurate long-term risk estimates for cardiac death and ischemic events beyond the risk calculations derived from clinical parameters and have corroborated its value as a cornerstone biomarker in estimating important patient outcomes [2–5]. Consequently, selective screening might identify at-risk patients potentially benefiting from early aggressive preventive measures [6].

However, in light of the increasing use of computed tomography (CT) for CAC scoring, radiation dose exposure has become an issue of concern. CAC scores are commonly derived from electrocardiogram-gated, non-contrast-enhanced CT of the heart which typically confers an effective radiation dose exposure of around 1–1.5 millisieverts (mSv) [7]. Meanwhile, and in line with the principle of 'as low as reasonably achievable' (ALARA), radiation exposure from coronary CT angiography (CCTA) has decreased significantly over the last decade thanks to the introduction of innovative scanning protocols and iterative reconstruction algorithms enabling lower tube voltage and current [8, 9]. Using state-of-the-art dose reduction methods, radiation dose exposure from routine contrast-enhanced CCTA in some centers nowadays consistently ranges in the sub-millisievert range [10]. Several approaches have been proposed to also lower the radiation dose exposure conferred by CT scans for CAC scoring. While some studies have, to this aim, focused on lowering the tube current [11], a promising approach lies in the reduction of the tube voltage as this yields a more profound reduction of radiation exposure [12, 13]. However, lowering the tube voltage harbors difficulties as tissue attenuation is closely related to photon energy, thus rendering the established thresholds for calculating CAC scores (i.e., Agatston scores) unsuitable if tube voltages other than the standard 120 kilovolt-peak (kVp) are applied [14]. In an attempt to overcome this problem, tube voltage-adapted thresholds for the calculation of CAC scores at lower tube voltages of 70 and 80 kVp have been developed. In a pilot study, applications of these thresholds have demonstrated a good agreement with CAC scores obtained from 70 and 80 kVp scans as compared to those obtained from standard 120 kVp scans while conferring less than a third of the radiation dose exposure (i.e., 0.12 and 0.19 mSv for 70 and 80 kVp scans vs. 0.60 mSv for the 120 kVp scans, respectively) [15]. However, tube current was intentionally kept identical for all scans [i.e., 200 milliamperes (mA)] in order to prevent implementing an additional confounder.

The primary aim of the present study was to evaluate further the accuracy of risk stratification derived from CAC scores from low kVp-scans in a larger, all-comer population and the secondary aim was to assess the impact of noise normalization, achieved through a patient-specific increase of tube current at lower tube voltages.

## Materials and methods

### Patient population

We prospectively included 170 consecutive adult patients without a history of revascularization or intracardiac devices who were referred for clinically indicated CAC scoring. One patient with a history of valve surgery had to be excluded due to artifacts caused by sternal cerclages. Thus, 169 patients were included in the final analysis. The study was approved by the local ethics committee (BASEC-NR. 2017-01158), and all patients provided written informed consent.

### Scan protocols, image reconstruction, and image analysis

All patients underwent the standard scanning protocol with 120 kVp and two additional scans with 80 kVp and 70 kVp immediately afterwards on a latest-generation 256-slice CT scanner (Revolution CT, GE Healthcare, Waukesha, MI, USA). For the standard 120-kVp scans, the tube current was fixed at 200 mA. In order to normalize noise and maintain a noise level comparable across all scans, the scanner-specific “noise index” algorithm was used to determine the tube current for the scans with lower tube voltages. In short, the noise index is referenced to the standard deviation of CT numbers within a region of interest in a water phantom of a specific size. A lookup table is then used to map the patient-specific attenuation values measured on the scout images to tube current values according to a proprietary algorithm [16]. All scans were performed in craniocaudal direction during inspiratory breath-hold with prospective electrocardiogram (ECG)-triggering. The scanning parameters included a collimation of 256 × 0.625 mm with a z-coverage of 12–16 cm. Gantry rotation time was 280 ms. Datasets with a displayed field of view of 25 cm with a slice thickness and an increment of 2.5 mm were reconstructed using filtered back projection. In every dataset, a region of interest (ROI) with a 20 mm diameter was placed in the aortic root at the level of the left main coronary artery on an axial image to measure mean attenuation (representing signal) and its standard deviation (SD) (representing noise) in Hounsfield units (HU). Values for effective radiation dose were calculated by multiplying the dose length product with a tube voltage-dependent conversion factor (i.e., 0.0145 mSv × mGray^−1^ × cm^−1^ for 120 kVp, and 0.0147 mSv × mGray^−1^ × cm^−1^ for 80 kVp and 70 kVp), as previously described [17].

### CAC scoring

**Fig. 1:**
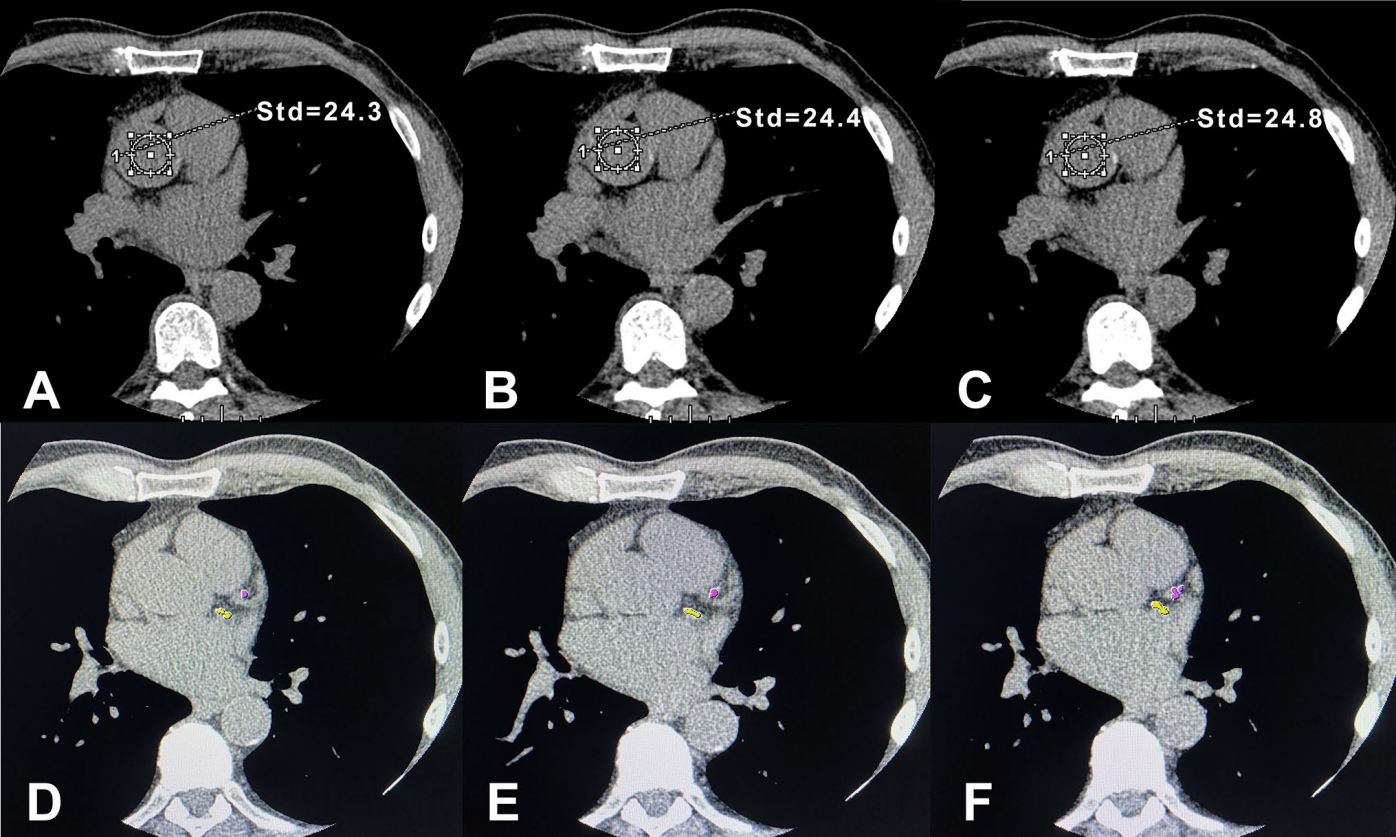
Image analysis example. Image analysis in a 63-year-old male patient with a body mass index of 21 kg/m^2^. The top row depicts noise measurements in the aorta for the 120-kVp (**A**), the 80-kVp (**B**), and the 70-kVp (**C**) scan with only minimal differences. The bottom row depicts CAC scoring in the 120-kVp (**D**), the 80-kVp (**E**), and the 70-kVp (**F**) scans. Calcifications in the left anterior descending (purple pixels) and in the left circumflex coronary artery (yellow voxels) were identified, resulting in a total CAC score of 198, 176, and 165 for the 120-kVp, the 80-kVp, and the 70-kVp scan, respectively

All datasets were transferred to a dedicated workstation (Advantage AW 4.4, GE Healthcare), running a prototype version of a semi-automatic software for CAC scoring (SmartScore 4.0, GE Healthcare), allowing for manual adjustment of the attenuation-based thresholds for CAC scoring. For the 80-kVp and 70-kVp CAC scoring, the novel thresholds (given in Table 1) were manually entered in the prototype software version, as previously described [15]. All pixels with attenuation equal or above the lowest threshold [e.g., ≥ 130 Hounsfield units (HU) for the standard 120-kVp scans] having an area ≥ 1 mm^2^ are automatically color marked, and lesions are manually selected by creating a ROI around all lesions found in a coronary artery (Fig. 1). The software then calculates the CAC score: In brief, a score for each ROI is calculated by multiplying the density score (i.e., 1–4, based on the scoring thresholds) and the area of calcifications. A total CAC score is then determined by adding up the scores of each CT slice. Of note, the thresholds for CAC scoring are only applied to pixels with a density equal to or larger than the lowest threshold and an area of ≥ 1 mm^2^, thus eliminating single pixels with values above the thresholds due to noise. Based on the CAC score, each patient was allocated to a risk class: 0, 1–10, 11–100, 101–400, and > 400. Risk-class changes for CAC scores derived from 80-kVp and 70-kVp scans were assessed, with the risk-class derived from standard 120-kVp scans serving as the risk-class reference. To provide an estimate of baseline variability, two experienced blinded readers additionally calculated CAC scores from the standard 120-kVp scans, and inter-reader reclassification rates were calculated.

**Table 1:** Novel kVp-adapted thresholds

Tube voltage (kVp)	Thresholds (HU) for lesion scores			
	1	2	3	4
120	130–199	200–299	300–399	≥ 400
80	177–271	272–408	409–544	≥ 545
70	207–318	319–477	478–636	≥ 637

Thresholds for 120 kVp are derived from Agatston et al. [14] and represent the standard of reference. Thresholds for 80 kVp and 70 kVp as proposed by Gräni et al. [15]

### Statistical analysis

Quantitative variables are expressed as the mean ± standard deviation or as median with interquartile range (IQR) if not normally distributed. Categorical variables are expressed as frequencies or percentages. The data were tested for normal distribution using the Kolmogorov–Smirnov test. Furthermore, the measurement of agreement between the different scans with regard to risk classification was tested using Kendall’s tau-b (Τ_b_) and Kappa (κ) statistics [18]. Supplementary, absolute CAC scores as derived from 80-kVp and 70-kVp scans were compared with standard 120-kVp scans using Bland–Altman (BA) analysis and a combination of intraclass correlation (ICC, absolute-agreement, 2-way mixed-effects models) estimates and linear regression for visualization. Sub-analyses were performed for patients with a body mass index (BMI) < 30 kg/m^2^ and BMI < 25 kg/m^2^. Image noise was compared using BA analysis. SPSS 25.0 (IBM Corporation, Armonk, NY, USA) was used for statistical analysis. A p-value of < 0.05 was considered statistically significant.

## Results

Patients' baseline characteristics are given in Table 2. Scan parameters, signal, and image noise are given in Table 3. Application of the noise index algorithm led to near-perfect normalization of noise for both the 80-kVp and 70-kVp scans as compared to the standard 120-kVp scans, with BA analysis revealing only minimal bias of − 0.4 HU and + 0.6 HU and narrow BA limits of agreement of − 3.0 to 2.2 HU and of − 2.5 to 3.7 HU, respectively. Median CAC score obtained from standard 120-kVp scans was 42 (IQR 0–199), and 49 (29%) patients presented with a CAC score of zero.

**Table 2:** Patient characteristics (*n* = 169)

Male gender	144 (85.2)
Age (years)	60 ± 9.0
Body weight (kg)	80 [71–88] range 47–150
Body size (cm)	175 [169–181] range 150–200
BMI (kg/m^2^)	26.1 [23.7–28.3] range 18.9–39.9
Cardiovaskular risk factors	
Smoking	45 (26.6)
Diabetes mellitus	14 (8.3)
Hypertension	68 (40.2)
Dyslipidaemia	71 (42.0)
Positive family history	45 (26.6)
Clinical symptoms	
Asymptomatic	46 (27.2)
Typical Angina pectoris	30 (17.8)
Atypical chest pain	49 (29.0)
Dyspnoe	39 (23.1)
Syncope	5 (3.0)
Medication	
Antiplatelet therapy	33 (19.5)
Beta-Blocker	30 (17.8)
ACI/ARB	51 (30.2)
Statin	48 (28.4)
CAC score risk class^a^	
0	49 (29.0)
1–10	18 (10.7)
11–100	45 (26.6)
101–400	34 (20.1)
> 400	23 (13.6)

Values given are mean ± standard deviation or median and interquartile range in square brackets or absolute numbers and percentages in brackets

*BMI* body mass index, *ACI/ARB* angiotensin-converting enzyme-inhibitor/angiotensin-receptor antagonist, *CAC* coronary artery calcium

^a^Based on CAC scoring obtained from standard 120-kVp CT scans

**Table 3:** Image noise and signal

	Peak tube voltage		
	120 kVp	80 kVp	70 kVp
Tube current (mA)	200 ± 0	460.1 ± 17.7	460.6 ± 18.3
Signal (HU)	48.5 ± 4.4	51.0 ± 5.5	53.1 ± 6.9
Noise (HU)	28.1 ± 3.7	27.7 ± 3.1	28.7 ± 2.8
SNR	1.8 ± 0.3	1.9 ± 0.3	1.9 ± 0.2

*HU* hounsfield units, *SNR* signal-to-noise ratio, *mA* milliamperes

**Fig. 2:**
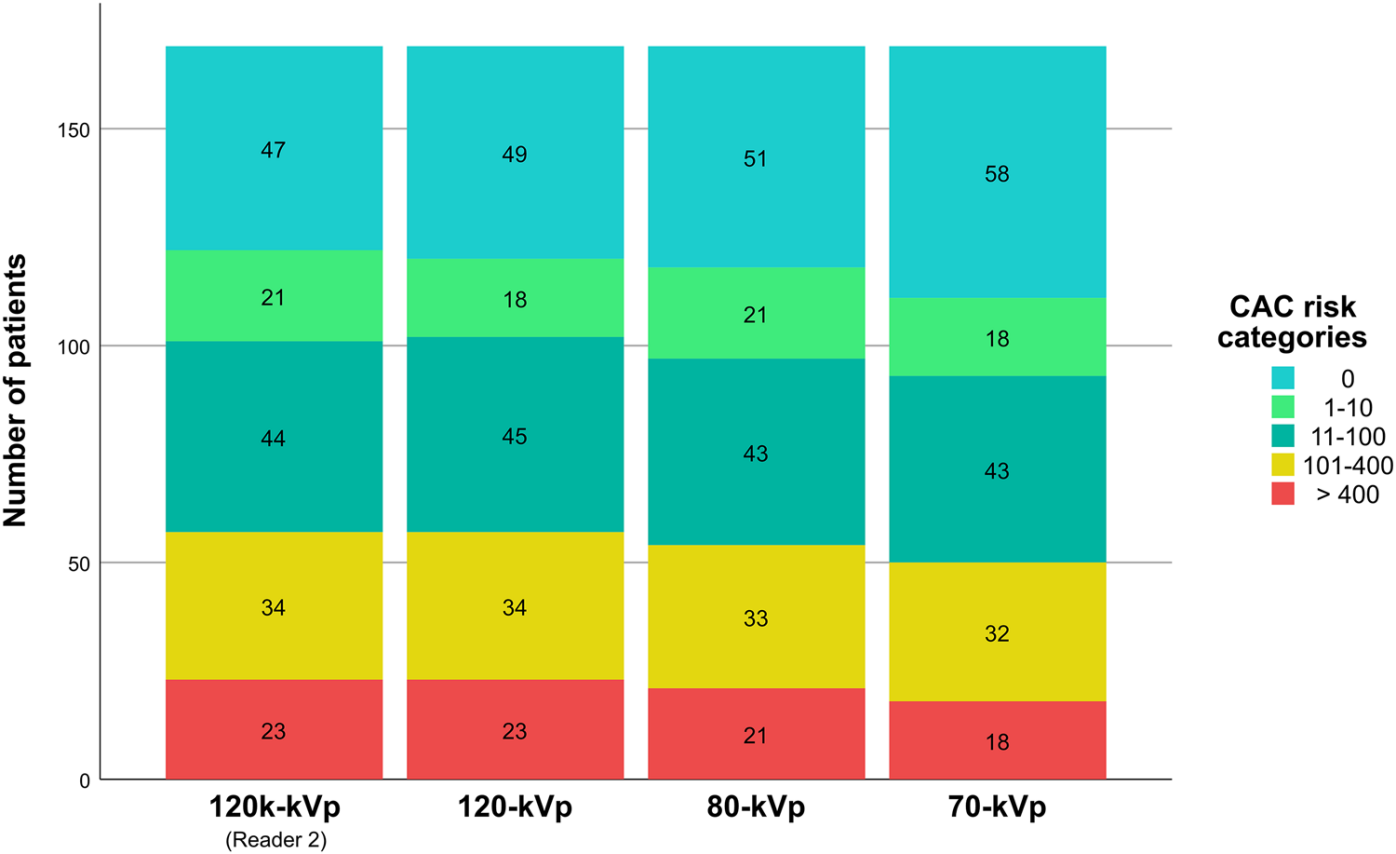
Visual representation of the risk stratification based on CAC scores derived from the 120-kVp (both readers), 80-kVp, and 70 kVp scans. The number of patients within each risk class is given within the boxes

In the overall population, risk reclassification could be observed in 12 (7.1%) and 29 (17.2%) patients for the 80-kVp and 70-kVp scans, respectively (Fig. 2; Tables 4, 5). With the exception of one patient, all reclassifications occurred towards lower risk classes. Regarding the inter-reader agreement, risk reclassification between readers was observed in 7 (4.1%) patients (Table 6).

**Table 4:** Agreement of CAC score-based risk classification derived from 80-kVp scans as compared with standard 120-kVp scans

			120 kVp		
CAC score	0	1–10	11–100	101–400	> 400
80 kVp					
0	**49**	2	0	0	0
1–10	0	**16**	5	0	0
11–100	0	0	**40**	3	0
101–400	0	0	0	**31**	2
> 400	0	0	0	0	**21**

Measure of agreement Kendall’s Τ_b_ = 0.967 and κ = 0.908, (both p < 0.001)

Bold incicates patients without risk reclassification

**Table 5:** Agreement of CAC score-based risk classification derived from 70-kVp scans as compared with standard 120-kVp scans

			120 kVp		
CAC score	0	1–10	11–100	101–400	> 400
70 kVp					
0	**49**	6	3	0	0
1–10	0	**12**	6	0	0
11–100	0	0	**36**	7	0
101–400	0	0	0	**26**	6
> 400	0	0	0	1	**17**

Measure of agreement Kendall’s Τ_b_ = 0.915 and κ = 0.777, (both p < 0.001)

Bold incicates patients without risk reclassification

**Table 6:** Inter-reader agreement of CAC score-based risk classification derived from 120-kVp scans

			120 kVp (Reader 1)		
CAC score	0	1–10	11–100	101–400	> 400
120 kVp (Reader 2)					
0	**47**	0	0	0	0
1–10	2	**17**	2	0	0
11–100	0	1	**43**	0	0
101–400	0	0	0	**33**	1
> 400	0	0	0	1	**22**

Measure of agreement Kendall’s Τ_b_ = 0.980 and κ = 0.947, (both p < 0.001)

Bold incicates patients without risk reclassification

In a sub-analysis of 140 (83%) patients who presented with a BMI < 30 kg/m^2^, changes in risk-class were observed in 10 (7.1%) and 19 (13.6%) patients for the 80-kVp and 70-kVp scans, respectively (Online Resources Tables 1, 2). In the 68 (40%) patients with a BMI < 25 kg/m^2^, reclassifications were observed in 2 (2.9%) and 5 (7.4%) patients for the 80-kVp and 70-kVp scans, respectively (Online Resources Online Tables 3, 4). Of note, no reclassifications were seen for CAC scores derived from the 80-kVp and 70-kVp scans in patients with a BMI below 24 kg/m^2^ and 21 kg/m^2^, respectively.

Results from BA analysis and ICC between absolute CAC scores calculated from the 80-kVp and 70-kVp scans using the novel thresholds and those derived from standard 120-kVp scans are provided in the Online Resources Figures S1 and S2. In brief, while the correlation was excellent for both the 80-kVp and the 70-kVp scans with an ICC of 0.966 and 0.958, respectively, BA limits of agreement widened with increasing CAC scores but narrowed down to − 19 to + 12 and − 30 to + 14 for CAC scores ≤ 100.

Mean effective radiation dose exposure from the 120-kVp, the 80-kVp, and the 70-kVp scans was 0.54 ± 0.03, 0.42 ± 0.02, and 0.26 ± 0.02 mSv.

## Discussion

Along the journey towards lower effective radiation dose exposure to patients undergoing CAC scoring, the present study extends our knowledge on the accuracy of cardiovascular risk stratification based on CAC scores in a setting of lowered tube voltage and application of adapted scoring threshold in a large all-comer population. We found that compared with standard 120 kVp/200 mA CAC scanning, lower peak tube voltages of 80 kVp and 70 kVp with a BMI-adapted tube current protocol led to a reduction of the mean effective radiation dose of up to 52% as compared to the standard 120-kVp protocol. We found a systematic underestimation of the CAC scores derived from the 80-kVp and 70-kVp scans as compared to the standard 120-kVp protocol, particularly for higher CAC scores and a strong dependency of the reclassification rate on the patients’ BMI and reclassifications occurring predominantly in patients with low CAC scores. Risk reclassification rates ranged between 7.1% and 17.2% for the overall population but were reduced to 2.9% and 7.4% in patients with a BMI below 25 kg/m^2^ and were not observed in patients with a BMI below 24 kg/m^2^ and 21 kg/m^2^ for the 80-kVp and 70-kVp scans, respectively.

To put these results into perspective, it is mandatory to take into consideration the interscan reproducibility inherent to CAC scoring, predetermining the minimal expected variability and risk misclassification rate of CAC scoring from repeated CT scans. In a comprehensive study from the Multi-Ethnic Study of Atherosclerosis (MESA) [19], the authors documented a CAC-adjusted repeatability limit of 4.2% (i.e., the value less than or equal to which the absolute difference between two test results obtained under repeatability conditions may be expected to be within a probability of 95%). Furthermore, it is known that even small variations in patient position on the scanner table may lead to substantially different Agatston scores with median absolute percentage differences of up to 147% and misclassification of approximately 10% of patients, particularly in patients with low CAC scores [20]. Additionally, Willemink et al. have demonstrated that the use of scanners from different vendors may lead to a risk reclassification in up to 6.5% of patients [21].

Against this background, the risk reclassification rates found for patients with a lower BMI in the present study are well in line with the inherent suboptimal reproducibility of CAC scoring. Even without taking into consideration repeat scanning, in our study, the inter-reader variability in a single scan led to a risk reclassification of 4.1% of patients. Hence, the error introduced by the kV-adapted thresholds for CAC scoring is unlikely to be excessive. However, they undeniably remain imperfect, as evidenced by the increasing reclassification rates with increasing BMI. Of note, a similar dependency was already documented in the pilot study first proposing the kVp-adapted scoring thresholds for CAC scans acquired with lower than 120-kVp scans by Gräni et al. [15]. In this study, reclassification rates ranged between 6.8% and 16.5% in the overall population but were strongly dependent on the BMI. Notably, however, in the study by Gräni et al., tube current was kept identical for all scans, a limitation deliberately accepted so as not to add another confounder when attempting to validate the novel thresholds. It may be hypothesized that the increase in noise introduced by lowering the tube voltage had negatively impacted the accuracy of CAC scores obtained from these low-dose CAC scans, particularly in patients with a higher BMI. The results from the present study deny this hypothesis because they are very well comparable to those from Gräni et al. but were obtained in a setting of normalized noise.

## Conclusions

Several conclusions can be drawn from the results of this study. First of all, CAC scoring with reduced tube voltage allows for accurate risk stratification if kVp-adapted thresholds for calculation of CAC scores are applied. Risk reclassifications rates for the lower kVp-scans are acceptable when considering the inherent interreader variability and predominantly occur in patients with a low extent of calcification. Secondly, reclassification rates were dependent on BMI, with no risk reclassifications for the 80-kVp and 70-kVp scans occurring in patients with a BMI below 24 kg/m^2^ and 21 kg/m^2^, respectively. Hence, our results corroborate the findings of a previous pilot study and underline the suggestion of applying a BMI-adapted scanning protocol using low-kVp scans in patients with a lower BMI but *without* adaptation of the tube current, thereby fully exploiting the dose reduction capabilities of low-kVp CAC scanning with adapted scoring thresholds. The results of the present study in conjunction with the findings from the pilot study by Gräni et al. suggest the use of a fixed tube current but BMI-adapted peak tube voltage approach in daily clinical routine where 120-kVp, 80-kVp, and 70-kVp scans are used for patients with a BMI ≥ 25 kg/m^2^, < 24 kg/m^2^, and < 21 kg/m^2^, respectively, offering a radiation dose reduction of up to 80% in selected patients with a lower BMI.

Dose reduction remains a crucial challenge in light of the fact that CAC scoring has been recognized as a valuable tool for risk stratification and that the number of examinations is constantly increasing. Any reduction of radiation exposure may translate into a reduced radiation-related risk not only on a patient but also on a population level. Moreover, as artificial intelligence (AI)-based CAC scoring tools are currently entering the clinical arena, expanding the application of CAC scoring to non-cardiovascular imaging (e.g., oncology) [22, 23], the availability of scoring thresholds for use in CT scans acquired with tube voltages other than 120 kVp is becoming strikingly relevant, as are the knowledge and awareness about the shortcomings of the currently available thresholds. Further studies are needed to refine the latter and to validate and further establish robust strategies for a reduction of radiation dose in CAC scoring, paving the way for its use as a potential screening application.

### Limitations

It may be perceived as a limitation that we have included consecutive all-comer patients, leading to a substantial proportion of individuals with a zero CAC score in the present cohort. However, this reflects a real-world population that may be encountered in clinical routine. Secondly, the definition of risk classes may seem arbitrary and are more nuanced than in some other studies. However, we felt that the comparability with a previous pilot study must be ensured.

### Supplementary Information

Below is the link to the electronic supplementary material.

Supplementary file1 (DOCX 18 kb)

Supplementary file2 (TIF 4630 kb). Figure S1: Linear regression analysis (A) and Bland-Altman plot (B) comparing CAC scores derived from 80-kVp scans to standard 120-kVp scans in all patients (n = 169). Bland-Altman limits of agreement were -122 to 76

Supplementary file3 (TIF 4173 kb). Figure S2: Linear regression analysis (A) and Bland-Altman plot (B) comparing CAC scores derived from 70-kVp scans to standard 120-kVp scans in all patients (n = 169). Bland-Altman limits of agreement were -261 to 159

## Abbreviations

CAC: Coronary artery calcium
CCTA: Coronary CT angiography
kVp: Kilovolt-peak

## Declarations

### Statistics and biometry

No complex statistical methods were necessary for this paper.

### Study subjects or cohorts overlap

The authors state that there is no overlap with other cohorts.

### Methodology

Prospective; Diagnostic study; Performed at one institution.
