# Supplementary material for: Risk stratification using coronary artery calcium scoring based on low tube voltage computed tomography
Source: Int J Cardiovasc Imaging. 2022 Apr 23;38(10):2227–34. doi: 10.1007/s10554-022-02615-x (PMC10509109; doi:10.1007/s10554-022-02615-x)
Supplement: Supplementary file 1 — Supplementary file1 (DOCX 18 kb) [file 10554_2022_2615_MOESM1_ESM.docx]

**Supplement Table 1 Agreement of CAC score-based risk classification derived from 80-kVp scans as compared with standard 120-kVp scans in patients with BMI <30 kg/m^2^ (*n* = 140)**

|  | **120 kVp** | | | | | |
| --- | --- | --- | --- | --- | --- | --- |
| **80 kVp** | CAC score | 0 | 1-10 | 11-100 | 101-400 | >400 |
|  | 0 | **40** | 1 | 0 | 0 | 0 |
|  | 1-10 | 0 | **15** | 5 | 0 | 0 |
|  | 11-100 | 0 | 0 | **35** | 2 | 0 |
|  | 101-400 | 0 |  | 0 | **23** | 2 |
|  | >400 | 0 |  | 0 | 0 | **17** |

Measure of agreement Kendall’s Τ_b_ = 0.967 and κ = 0.908, Kendall-Tau-b = 0.967 (both p<0.001).

**Supplement Table 2 Agreement of CAC score-based risk classification derived from 70-kVp scans as compared with standard 120-kVp scans in patients with BMI <30 kg/m^2^ (*n* = 140)**

|  | **120 kVp** | | | | | |
| --- | --- | --- | --- | --- | --- | --- |
| **70 kVp** | CAC score | 0 | 1-10 | 11-100 | 101-400 | >400 |
|  | 0 | **40** | 4 | 2 | 0 | 0 |
|  | 1-10 | 0 | **12** | 4 | 0 | 0 |
|  | 11-100 | 0 | 0 | **34** | 3 | 0 |
|  | 101-400 | 0 | 0 | 0 | **21** | 5 |
|  | >400 | 0 | 0 | 0 | 1 | **14** |

Measure of agreement Kendall’s Τ_b_ = 0.930 and κ = 0.824 (both p<0.001).

**Supplement Table 3 Agreement of CAC score-based risk classification derived from 80-kVp scans as compared with standard 120-kVp scans in patients with BMI <25 kg/m^2^ (*n* = 68)**

|  | **120 kVp** | | | | | |
| --- | --- | --- | --- | --- | --- | --- |
| **80 kVp** | CAC score | 0 | 1-10 | 11-100 | 101-400 | >400 |
|  | 0 | **23** | 0 | 0 | 0 | 0 |
|  | 1-10 | 0 | **8** | 1 | 0 | 0 |
|  | 11-100 | 0 | 0 | **18** | 0 | 0 |
|  | 101-400 | 0 | 0 | 0 | **10** | 1 |
|  | >400 | 0 | 0 | 0 | 0 | **7** |

Measure of agreement Kendall’s Τ_b_ = 0.988 and κ = 0.961 (both p<0.001).

**Supplement Table 4 Agreement of CAC score-based risk classification derived from 70-kVp scans as compared with standard 120-kVp scans in patients with BMI <25 kg/m^2^ (*n* = 68)**

|  | **120 kVp** | | | | | |
| --- | --- | --- | --- | --- | --- | --- |
| **70 kVp** | CAC score | 0 | 1-10 | 11-100 | 101-400 | >400 |
|  | 0 | **23** | 1 | 0 | 0 | 0 |
|  | 1-10 | 0 | **7** | 2 | 0 | 0 |
|  | 11-100 | 0 | 0 | **17** | 0 | 0 |
|  | 101-400 | 0 | 0 | 0 | **9** | 1 |
|  | >400 | 0 | 0 | 0 | 1 | **7** |

Measure of agreement Kendall’s Τ_b_ = 0.968 and κ = 0.903 (both p<0.001).
